# Temporal-Spatial Transcriptome Analyses Provide Insights into the Development of Petaloid Androecium in *Canna indica*

**DOI:** 10.3389/fpls.2016.01194

**Published:** 2016-08-17

**Authors:** Xueyi Tian, Qianxia Yu, Huanfang Liu, Jingping Liao

**Affiliations:** ^1^Key Laboratory of Plant Resources Conservation and Sustainable Utilization, South China Botanical Garden, Chinese Academy of SciencesGuangzhou, China; ^2^College of Life Sciences, University of Chinese Academy of SciencesBeijing, China

**Keywords:** transcriptome, RNA-Seq, petaloid staminode, Zingiberales, *Canna indica*, ABC model

## Abstract

*Canna indica* (Zingiberales) is one of the most important ornamental species characterized with beautiful petaloid staminodes, which are considered to evolve from stamens. However, the genetic basis for the development of petaloid staminodes remains unclear largely because the genomic sequences are not available. By using RNA-Seq, we sequenced the transcripts in the flower of *C. indica*, and quantified the temporal gene expressions in flower primordium and differentiated flower, as well as the spatial gene expressions in petal and petaloid staminode. In total, 118,869 unigenes were assembled, among which 67,299 unigenes were annotated. Quantification analysis identified the differentially expressed genes in the temporal and spatial two comparisons, based on which, Gene Ontology enrichment analysis highlighted the representative terms in each sample, such as specification of organ number in flower primordium, growth in differentiated flower, secondary cell wall biogenesis in petal and cell division in petaloid staminode. Among the 51 analyzed MADS-box unigenes, 37 were up-regulated in differentiated flower compared with those in flower primordium. A-class unigenes were expressed higher in petal than in petaloid staminode, and C-class unigenes were expressed oppositely, whereas B-class unigenes demonstrated close expression levels in these two organs, indicating that petaloid staminode retains stamen identity to some degree. *In situ* hybridization provided more detailed expression patterns of these unigenes, and revealed the extended expression of B-class to the carpel at later stages when the style turned flat. These results constitute a preliminary basis for the study of flower development in *C. indica* and can be applied in further study of the evolution of Zingiberales.

## Introduction

The Zingiberales is an order of tropical monocots comprising eight families (Tomlinson, 1962; Dahlgren and Rasmussen, 1983). Considerable species in Zingiberales are widely cultivated, including banana, ginger, and many kinds of ornamental plants. The main line of the evolution of Zingiberales is the number of fertile stamen decreasing by (6)5 → 1 → 1/2, and the number of petaloid organs increasing contrarily. Based on the stamen number, the eight families in Zingiberales are often divided into two groups: paraphyletic banana group (Musaceae, Lowiaceae, Strelitziaceae, and Heliconiaceae) with five (occasionally six) fertile stamens, and monophyletic ginger group (Zingiberaceae, Costaceae, Cannaceae, and Marantaceae) with one or a half fertile stamen (Tomlinson, 1962; Kress, 1990; Rudall and Bateman, 2004). Bearing only a half fertile stamen in the flower, *Canna indica* (Cannaceae) is regarded as an ideal material to investigate the phylogenetic evolution of Zingiberales.

In the flower of *C. indica*, the fertile stamen consists of a one-theca anther and a petaloid appendage, while the other androecial members are all completely petaloid structures (Kirchoff, 1983; Miao et al., 2014). As implied by the name, petaloid staminodes are thought to have evolved from stamens. Recently, many efforts have been made to reveal the molecular basis for androecial petaloidy of Zingiberales species including *C. indica*, yet the exact mechanism remains unclear (Bartlett and Specht, 2010; Song et al., 2010; Almeida et al., 2013; Yockteng et al., 2013; Fu et al., 2014; Almeida et al., 2015).

In core eudicots such as *Arabidopsis*, the ABC model has provided a rational interpretation for floral organ specification (Bowman et al., 1991; Coen and Meyerowitz, 1991; Weigel and Meyerowltzt, 1994). According to this model, A-class genes *APETALA1* (*AP1*) and *AP2* confer sepal identity in the first floral whorl; A-class genes together with B-class genes *AP3* and *PISTILLATA* (*PI*) specify petal identity in the second whorl; stamen identity in whorl 3 is determined by *AP3, PI* and the C-class gene *AGAMOUS* (*AG*), while the expression of the *AG* alone in whorl four promotes carpel development. Mutual repression between the A- and C-functions is an essential postulate of ABC model. With the exception of *AP2*, the ABC genes encode MADS domain proteins. However, the classic ABC model could not be perfectly applied to all clades, and researches have revealed the limited applicability of A-functions. In grasses such as maize, rice, and wheat, the B- and C-class genes are functionally conserved with eudicots (Mena et al., 1996; Kang et al., 1998; Ambrose et al., 2000; Munster, 2001; Meguro et al., 2003), while no clear evidence has been found in these species to support the role of A-class genes in specifying petal identity, neither in other species besides *Arabidopsis* (Causier et al., 2010). In addition, besides in *Arabidopsis*, none of the described *ap1* or *ap2*-like mutants shows ectopic C-function in the perianth (Litt, 2007). Recently, a new (A)BC model with more widespread applicability was proposed. In this model, the (A)-function genes, including but not limited to *AP1* and *AP2*, fulfill several roles, such as establishing floral meristem identity and promoting the production of sepals, which serve as the ground state of floral organs. With the regulation of (A)-function genes, B- and C-functions genes act to specify organ identities. Thus, the identity of whorl 2 is determined by B-class genes, while whorl 3 and whorl 4 are specified in a similar manner to the classical ABC model (Causier et al., 2010).

In Zingiberales, petaloid staminodes replace stamens in four of the eight families (Kirchoff et al., 2009). According to the ABC model (whether classic or updated), it is hypothesized that this kind of homeotic conversion is resulted from the absence of C-class genes in these petaloid staminodes (Wake et al., 2011). However, evidences in *Alpinia hainanensis* (Zingiberaceae) and *C. indica* have shown that C-class genes were still expressed in the petaloid androecial members (Song et al., 2010; Almeida et al., 2013; Fu et al., 2014). Furthermore, expressions of B-class *GLOBOSA* (*GLO*, homologous to *PI*)-like genes were not confined within corolla and androecium (Bartlett and Specht, 2010), making it more complicated to explain the organ identity specification in *C. indica* as well as in Zingiberales.

In recent years, next generation sequencing (NGS) techniques have provided fascinating opportunities in life sciences and remarkably improved the efficiency of gene discovery. In Zingiberales, D'Hont et al. (2012) described the genome sequence of *Musa acuminata* (Musaceae), providing a crucial basis for genetic improvement of banana. Besides, transcriptome sequencing has facilitated various kinds of studies on Zingiberales species such as molecular marker development and disease-resistant researches (Karthika et al., 2015; Ravishankar et al., 2015). As for flower development, no complete transcriptome analysis has been reported in Zingiberales except for some fragmentary descriptions on expression levels of several genes.

In this study, transcriptome sequencing of *C. indica* flower and quantitative gene expressions in different stages and different floral organs were performed using RNA-Seq. Comprehensive information about gene sequences, expressions and functions were provided, which could facilitate our understanding of the molecular mechanisms of flower development in *C. indica* and the relative species. Based on these data, we analyzed the expressions of ABC genes together with *in situ* hybridization approach, providing fundamental insights into the flower development of *C. indica*.

## Materials and methods

### Plant materials

*Canna indica* plants were grown in South China Botanical Garden (SCBG, Guangzhou), and the flower materials were collected during May and June.

### Scanning electron microscopy (SEM)

Young inflorescences were fixed in FAA (90 parts 70% ethyl alcohol: 5 parts glacial acetic acid: 5 parts 40% formaldehyde). Bracts and larger floral organs were removed under a dissecting microscope; then the inflorescences were dehydrated in an alcohol series (75, 85, 95, 100, and 100%) and transferred to isoamyl acetate. The materials were critical point dried with CO_2_, mounted on stubs, gold-coated in the JFC-1600 Auto Fine Coater (JEOL, Tokyo, Japan), and observed under a JSM-6360LV SEM (JEOL) operated at 10 kV.

### Transcriptome sequencing

Two transcriptome libraries were constructed from four flower samples: sample 1 (flower primordia), sample 2 (young flowers of 1–2 mm in length), sample 3 (young flowers of 3–10 mm in length), and sample 4 (flowers of 1–4 cm in length). Total RNA of these four samples were extracted using TruSeq™ RNA Sample Preparation Kit (Illumia, SanDiego). One sequencing library (flower primordium/FP) was constructed with the RNA of sample 1, while RNA from sample 2, 3, and 4 were equally mixed for the construction of a second library (differentiated flower/DF). After DNase I treatment, magnetic beads with Oligo (dT) were used to isolate mRNA, which was then fragmented into short fragments by mixing with the fragmentation buffer. The cDNA was synthesized using the mRNA fragments as templates. Short fragments were purified and resolved with EB buffer for end reparation and single nucleotide A (adenine) addition. After that, the short fragments were connected with adapters. The suitable fragments were selected for the PCR amplification as templates. Then Agilent 2100 Bioanaylzer and ABI StepOnePlus Real-Time PCR System were used in quantification and qualification of the sample libraries. At last, the libraries were sequenced using Illumina HiSeq™ 2000, and 90 nt raw reads were generated.

### Transcriptome assembly and annotation

The dirty raw reads [a. containing adapters, b. containing unknown nucleotides larger than 5%, c. low quality reads which the percentage of low quality bases (base quality ≤ 10) is more than 20%] were discarded, and the remaining clean reads in each library were assembled into one set of unigenes using Trinity (Grabherr et al., 2011), and then the two sets of unigenes were merged into a more complete set of unigenes, which served as the sequence reference in this research. The unigenes were divided into two classes. One was clusters with several unigenes between which the similarity was more than 70%, and the prefix is CL. The other class was singletons with the prefix of Unigene.

The unigene sequences were aligned by BLASTX to protein databases NR, Swiss-Prot, KEGG, and COG (*e* < 0.00001), and aligned by BLASTN to nucleotide databases NT (*e* < 0.00001) to get the annotations. Blast2GO program was used to get the GO annotation (Conesa et al., 2005), and WEGO software was used to do GO functional classification for all the unigenes (Ye et al., 2006). MADS-box unigenes were identified based on the Nr annotation. Besides the best hit, other alignments with relatively high blast scores were also referenced for the identification of putative ABC homologs.

### Gene expression analysis

A portion of the sequencing reads in FP and DF was used to quantify the temporal gene expression in these two samples. In addition, a spatial gene expression analysis in floral organs was also performed. Young petals (P) and petaloid staminodes (PS) about 1.5 mm in length were collected for RNA extraction when they could be carefully dissected. Procedures of library construction and sequencing were the same with those described above, except that the length of reads here was 49 nt.

Clean reads in FP, DF, P, and PS were mapped to the reference unigenes using SOAPaligner/SOAP2 (Li et al., 2009). No more than 2 mismatches were allowed in the alignment. The expression level for each gene is determined by the numbers of reads uniquely mapped to the specific gene (unique match), and was calculated by using RPKM method (Mortazavi et al., 2008). The differentially expressed unigenes (DEGs) between the two samples were identified using the DEGseq R package (1.12.0) (Wang et al., 2010), with the read counts as input. The raw read counts were previously adjusted by edgeR package through one scaling normalized factor (Robinson et al., 2010). The *p*-values were adjusted to *q*-values for multiple testing corrections (Storey and Tibshirani, 2003), and *q*-value of 0.001 and log2 (fold-change) of 1 were set as the threshold for significantly differential expression. GO enrichment analysis were using agriGO program with customized annotation (Du et al., 2010), and the GO terms with FDR less than 0.05 were considered significantly enriched in the DEGs.

### qRT-PCR

Total RNA was extracted from petals and petaloid staminodes of the same stages with those in RNA-Seq using TRIzol (Invitrogen) and then treated with DNase I (TaKaRa) followed by phenol/chloroform extraction to remove DNA contamination. Approximately 4 μg of purified RNA was used for reverse transcription with oligo(dT) primers. qRT-PCR was performed using specific primer pairs (Supplementary Table 1) with the MyiQ2 two-color real-time PCR detection system (Bio-Rad). Actin mRNA was used as an internal control, and the comparative threshold cycle (Ct) method was used to determine relative transcript levels of each tested genes. Three technical replicates were performed.

### *In situ* hybridization

Sections (7 μm thick) of young inflorescence without phyllary were prepared following pretreatment and hybridization methods described previously (Brewer et al., 2006). Digoxigenin (DIG)-labeled hybridization probes of *CiAP1-1* (457 nt), *CiGLO-1* (693 nt), and *CiAG-1* (675 nt) mRNA were prepared by *in vitro* transcription (Roche) according to the manufacturer's protocol. The primer pairs are listed in Supplementary Table 1.

## Results

### Flower development

**Figure 1:**
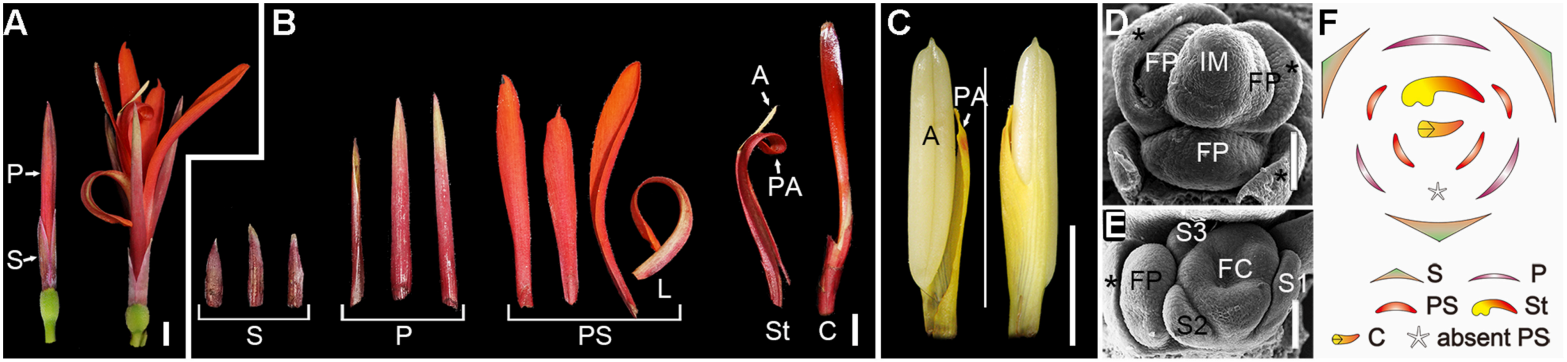
**Flower morphogenesis of *Canna indica*. (A)** A pre-anthesis flower (left) and mature flower at anthesis (right). **(B)** Dissected floral organs of a mature flower. **(C)** Adaxial (left) and abaxial (right) sides of a fertile stamen. **(D)** Initiation of floral primordia. Asterisk indicates bract or bractlet. **(E)** A flower pair (the two flowers in the axil of a bract) with a flower 1 at differentiated stage (right) and the primordium of flower 2 (left). **(F)** Schematic representation of the flower. S, sepal; P, petal; PS, petaloid staminode; L, labellum; St, stamen; A, anther; PA, petaloid appendage; C, carpel; IM, inflorescence meristem; FP, floral primordium; FC, floral cup. Bars = 5 mm in **(A–C)**, 100 μm in **(D,E)**.

The flower of *C. indica* comprises 3 sepals, 3 petals, 4, or 5 (occasionally 6) androecial members and 3 carpels (Figures 1A,B,F). Within the androecial whorl, the only fertile stamen comprises a one-theca anther and a petaloid appendage, while the other members are all petaloid staminodes, including a labellum (Figures 1B,C). Floral organogenesis of *C. indica* has been previously reported (Kirchoff, 1983; Almeida et al., 2013; Miao et al., 2014). Partially, due to the differentiation of the androecium, the flower development process of *C. indica* is quite complicated.

Flower primordia were initiated in the axils of bracts (Figure 1D). When the organogenesis process starts, sepals are initiated first, and then serve to protect the inner organs at early stages (Figure 1E). However, petals grow fast after initiation and soon surmount the sepals, and begin to act as the main protective organs instead of sepals till anthesis (Figure 1A). Indeed, except for their difference in size, sepals and petals share similarities in shape, color and texture, and both of them are much thicker and tougher than the inner flexible petaloid staminodes (Figure 1B). Petaloid staminodes are the most attractive parts in a mature flower of *C. indica*, and look similar to the bright-colored petals of other ornamental species rather than the petals of *Canna indica* itself. Although both are petal-like organs, petaloid staminodes and petals are different in size, shape, color, and texture (Figures 1A,B).

### Transcriptome sequencing and assembly

**Figure 2:**
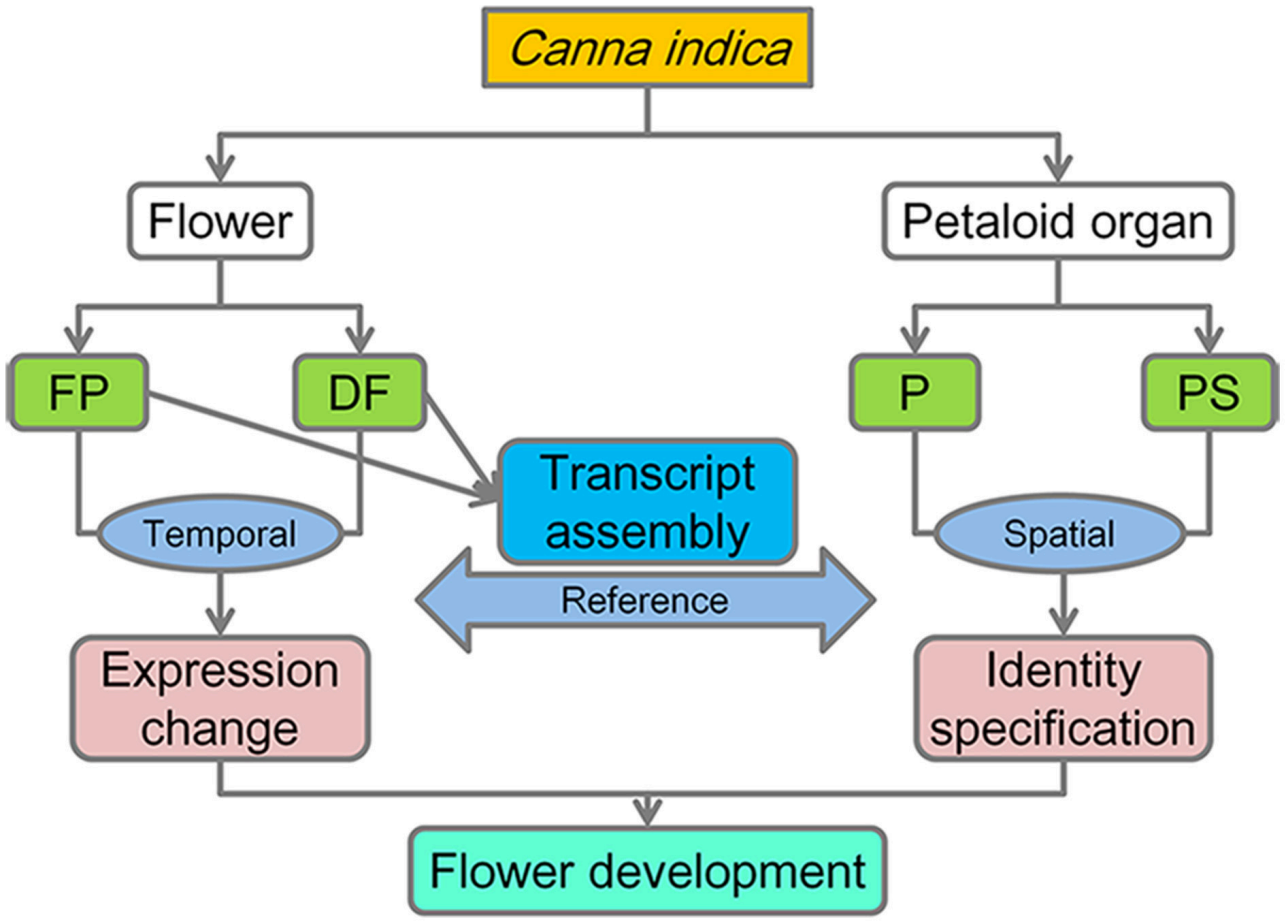
**Pipeline of the RNA-Seq analysis**.

In order to get a comprehensive understanding about the flower development process of *C. indica*, a temporal-spatial RNA-Seq analysis was carried out (Figure 2).

Since there is no genome reference for this species currently, a set of inclusive transcriptome assembly was needed. Flower development process concerns various stages from flower primordium initiation to anthesis, and we took flower primordium (FP) and differentiated flower (DF, included flowers at various developmental stages, see materials and methods) materials to perform transcriptome sequencing respectively. Ninety Nucleotide databases reads were generated and sequenced using Illumina HiSeq™ 2000 platform. In total, 118,693,512 (FP) and 116,509,018 (DF) raw sequencing reads were generated in each library. After cleaning and quality checks, the remaining 111,087,434 (FP) and 105,704,938 (DF) clean reads were assembled into two sets of unigenes, which were then merged to one set of 118,869 unigenes. The average length of all the unigenes was 1053 nt, and the N50 length was 1766 nt (Table 1). In addition, 44,076 unigenes were ≥1000 nt in length, 6719 unigenes were ≥3000 nt, and none was <200 nt.

**Table 1:** **Summary for floral transcriptome sequencing and assembly**.

**Unigene**	**Total number**	**Total length (nt)**	**Mean length (nt)**	**N50**	**Clusters**	**Singletons**
FP	112,984	127,816,607	1131	1931	42,117	70,867
DF	93,080	78,199,579	840	1332	32,099	60,981
All	118,869	125,202,958	1053	1766	43,615	75,254

**Figure 3:**
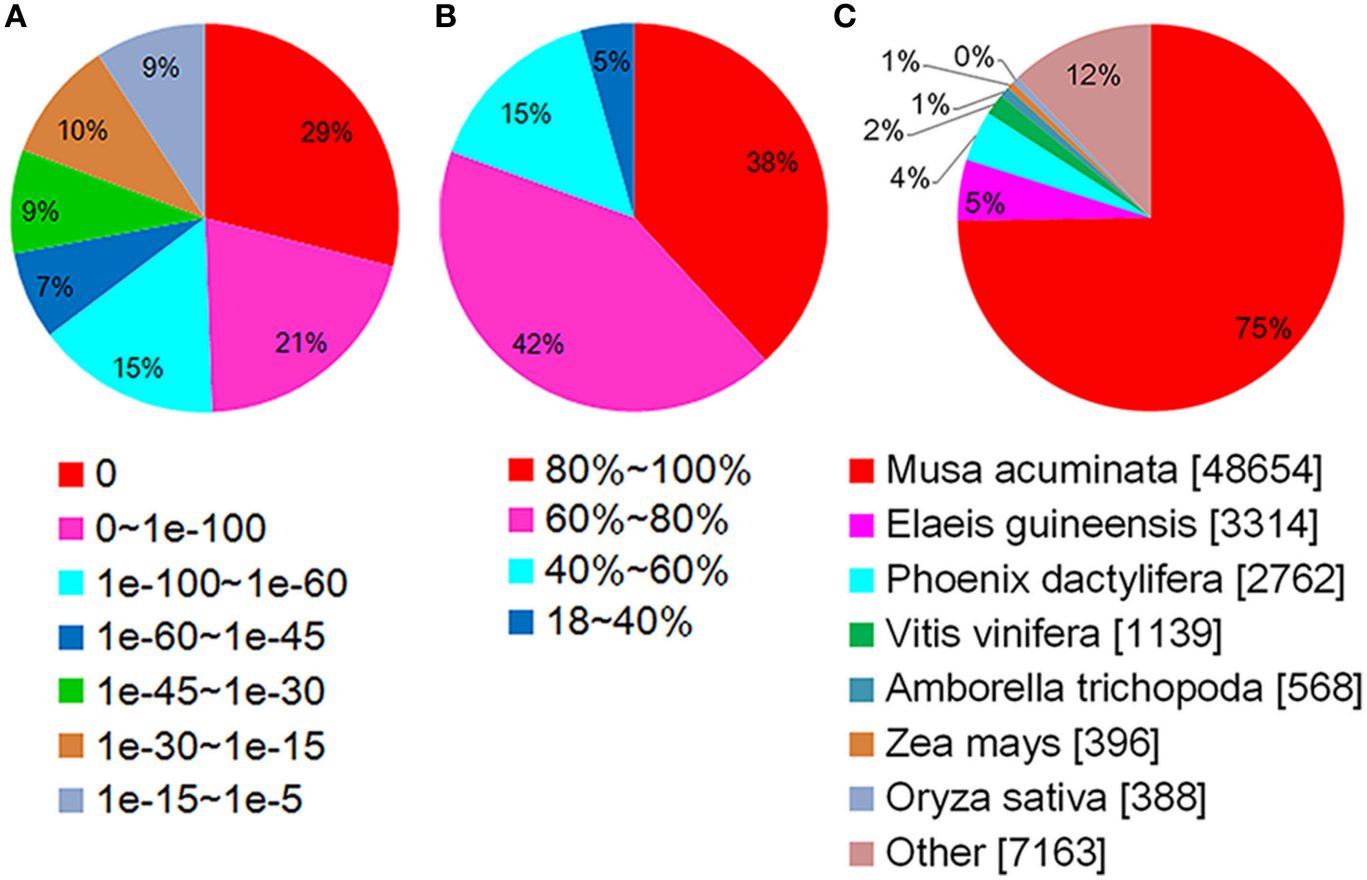
**Annotations of unigenes in Nr database**. **(A)** Statistics of *E*-value distribution. **(B)** Statistics of similarity distribution. **(C)** Statistics of species distribution.

**Figure 4:**
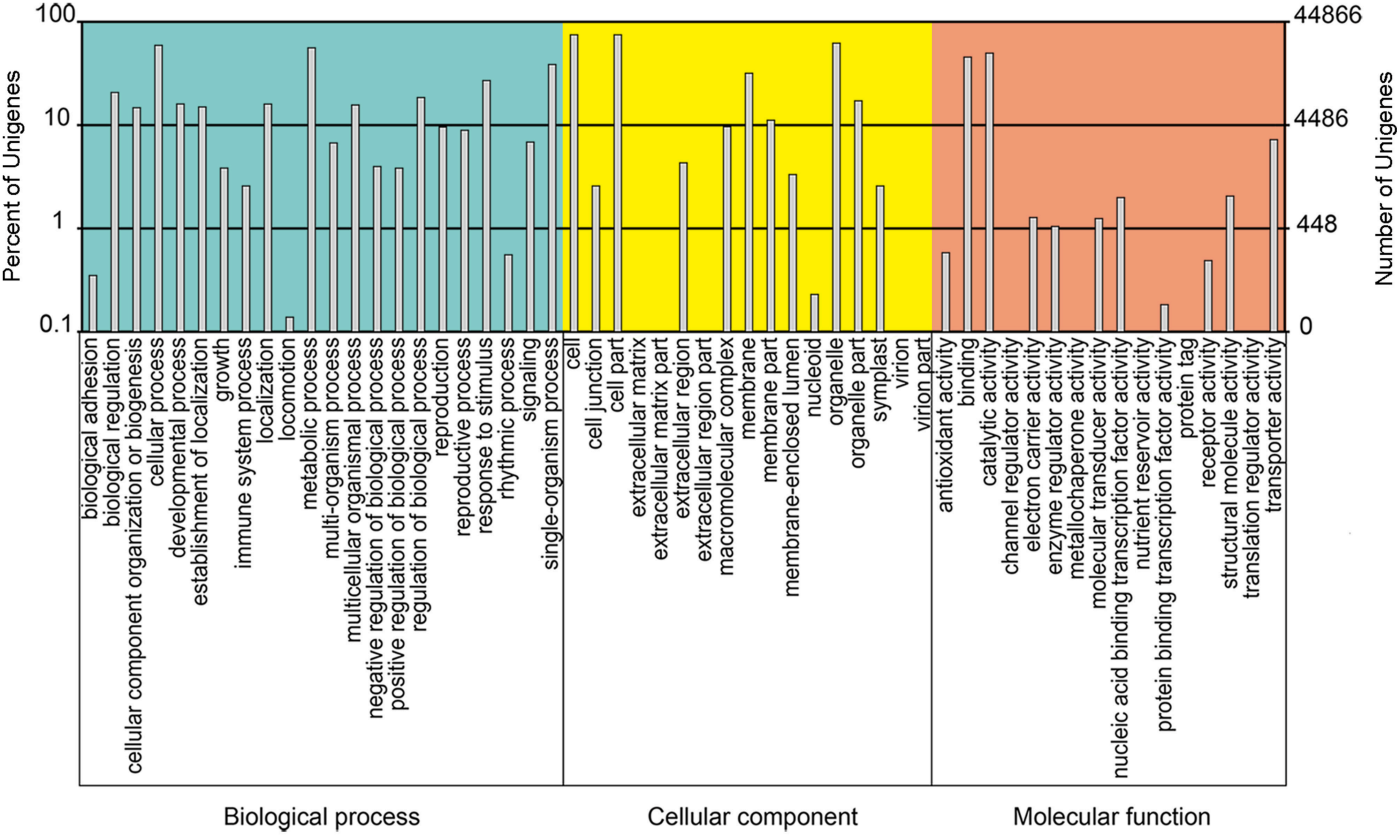
**GO classification of the annotated unigenes**.

Using BLASTX, the unigenes were aligned to the protein databases in the priority order of NR, NT, Swiss-Prot, KEGG, COG, and GO (*e* < 0.00001), and totally 67,299 unigenes were annotated. 65,392 unigenes were annotated to NR database, among which about 50% showed blast *e* < 1*e*-100 (Figure 3A), 80% shared high similarity (≥60%) with the homologs they were annotated to (Figure 3B). About 75% (48,654) of the annotated unigenes shared highest similarity with the genes of *Musa acuminate* (Zingiberales, Musaceae), and 5 and 4% with *Elaeis guineensis* and *Phoenix dactylifera* (Arecaceae), two Arecales species (Figure 3C). Twenty-six thousand five hundred and twenty-nine annotated unigenes were classified into 25 function classifications in COG database, and 38,318 were mapped to 128 KEGG pathways (data not shown). With GO annotation, 44,866 unigenes were categorized into 55 functional groups, which belong to three categories: molecular function, cellular component and biological process. Cellular process and metabolic process, cell and cell part, catalytic activity, and binding were the two dominant groups in each category respectively (Figure 4). These unigenes provided valuable sequence and annotation reference for the following quantification analysis.

### Quantitative temporal-spatial gene expression

**Figure 5:**
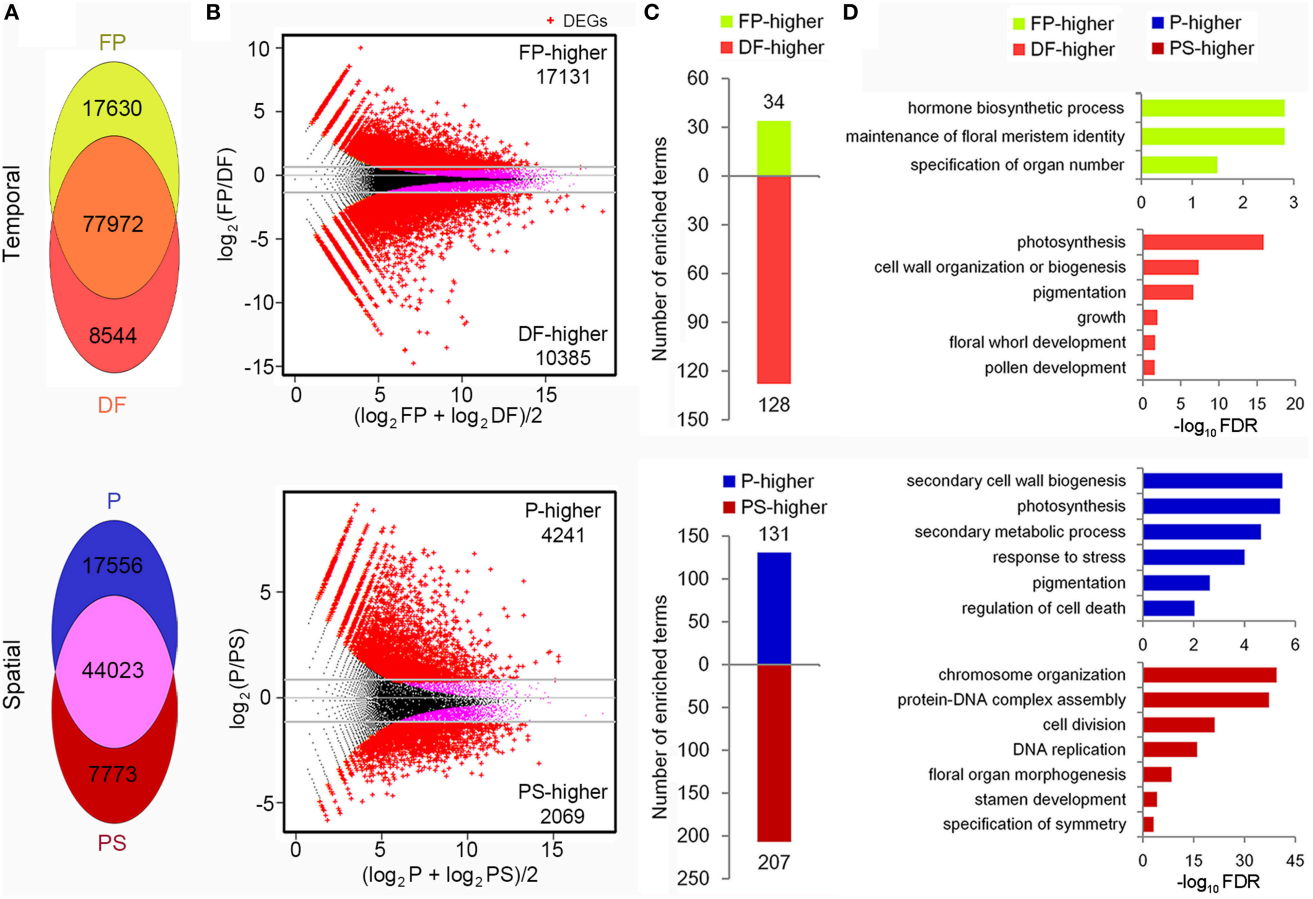
**Temporal-spatial expression and function analysis of unigenes**. **(A)** Distribution of detected unigenes in FP, DF and P, PS. **(B)** MA plots (log ratio vs. abundance) showing the DEG (red points) identification. **(C)** Numbers of the enriched biological process terms in each group of DEGs. **(D)** The representative terms in each group of DEGs.

Flower development process concerns various stages from flower primordium initiation to anthesis, and floral organ identity determination happens in FP while flower organ development mainly takes place in DF. Accordingly we took these two stages to perform temporal gene expression analysis, although DF represented various stages rather than a specific one (Figure 2). To quantify the expression of the unigenes, 36,393,804 and 36,749,397 sequencing reads in FP and DF were used to map to the reference assembly. The unigene expression levels were calculated with RPKM (Reads Per Kb per Million reads) method (Mortazavi et al., 2008). We set a threshold value at 0.3 RPKM to determine whether or not a gene was expressed in a specific sample. With this standard, 95,602 and 86,516 unigenes were expressed in FP and DF respectively. Seventeen thousand and six hundred and thirty and Eight thousand five hundred and forty-four unigenes were expressed specifically in FP and DF, and 77,972 unigenes were expressed in both the two stages (Figure 5A).

As the first step to investigate the genetic basis for the differences between petal (P) and petaloid staminode (PS), the global gene expression in these two organs was analyzed (Figure 2). Considering the differences in the organogenesis of these two organs, we took young petals and petaloid staminodes of about 1.5 mm in length as materials for quantitative RNA-Seq analysis. 49-nt reads were generated in each RNA sample which were subsequently sequenced for the library construction. After data filtering, the retained clean reads were used for mapping to the reference unigenes. The total numbers of clean reads in the two libraries were 11,779,727 and 11,668,376, of which 91.54 and 92.41% could be mapped to the reference. Seventy-five lakhs twelve thousand eight hundred and sixteen and Seventy-five lakhs fifty eight thousand five hundred and seventy-eight reads were mapped to a unique position (Table 2). In total, 78,255 unigenes were mapped in at least one organ. Not counting those with RPKM < 0.3, we got the expressions of 69,352 unigenes in P and PS. Sixty one thousand five hundred and seventy-nine and Fifty one thousand seven hundred and ninety six were expressed in P and PS respectively, with 17,556 specifically expressed in P and 7773 specifically in PS, and 44,023 in both organs (Figure 5A).

**Table 2:** **Sequencing quality evaluation and transcript alignments of the reads in P and PS**.

**Summary**	**P**	**PS**
Total reads	11,779,727	11,668,376
Total basepairs	577,206,623	571,750,424
Total mapped reads	10,782,916	10,782,484
Unique match	7,512,816	7,558,578
Multi-position match	3,270,100	3,223,906
Unmapped reads	996,811	885,892

### Function analysis of the differentially expressed unigenes

With DEGseq R package (*q* < 0.001, |log2 fold-change|≥1), the differentially expressed unigenes (DEGs) between FP and DF, and between P and PS were identified (Storey and Tibshirani, 2003; Wang et al., 2010). Compared with FP, 17,131 unigenes were down-regulated and 10,385 were up-regulated in DF, and these unigenes were defined as FP-higher and DF-higher DEGs respectively (Figure 5B). While in P-PS comparison, there were 6310 DEGs between the two organs, among which 4241 expressed higher in P (defined as P-higher DEGs) and 2069 expressed higher in PS (defined as PS-higher DEGs; Figure 5B).

To find out the representative biological processes in each sample, GO enrichment analysis were carried out based on the PF-higher, DF-higher, P-higher, and PS-higher DEGs. In PF-higher DEGs, 34 biological progress terms were enriched, among which, maintenance of floral meristem identity and specification of organ number explicitly presented the main biological processes in this stage (Supplementary Table 2; Figures 5C,D). While in DF-higher DEGs, 128 terms got enriched, far more than that in FP-higher DEGs (Figure 5C). Perhaps this was because a differentiated flower is more complicated than a flower primordium, whether in structure or function. The representative terms, such as pigmentation, growth, and floral whorl development gave a brief but clear description on the development processes in DF (Supplementary Table 2; Figure 5D).

In the P-higher DEGs, 131 biological process terms were enriched. Secondary cell wall biogenesis term indicated that cells in petals may possess thicker cell walls than those in petaloid staminodes, providing possible explanation that petals are tougher than petaloid staminodes; photosynthesis term indicated that as the outer whorl organs, young petals possess higher ability or potential to perform photosynthesis than petaloid staminodes in the inner whorls; in addition, response to stress term was probably related to the protection function of petals (Supplementary Table 2; Figures 5C,D). As for the PS-higher DEGs, chromosome organization, protein-DNA complex assembly and DNA replication were highly enriched (Supplementary Table 2; Figures 5C,D). It was conjectured that at the tested stage, young petaloid staminodes were experiencing rapid and continuous cell division, which was also an enriched term. In addition, stamen development term was also enriched in PS-higher DEGs, indicating that quite a few unigenes that participating in stamen development were also expressed in petaloid staminodes, and exhibited higher expressions in PS than P.

### Temporal-spatial expression patterns of MADS-box unigenes

**Figure 6:**
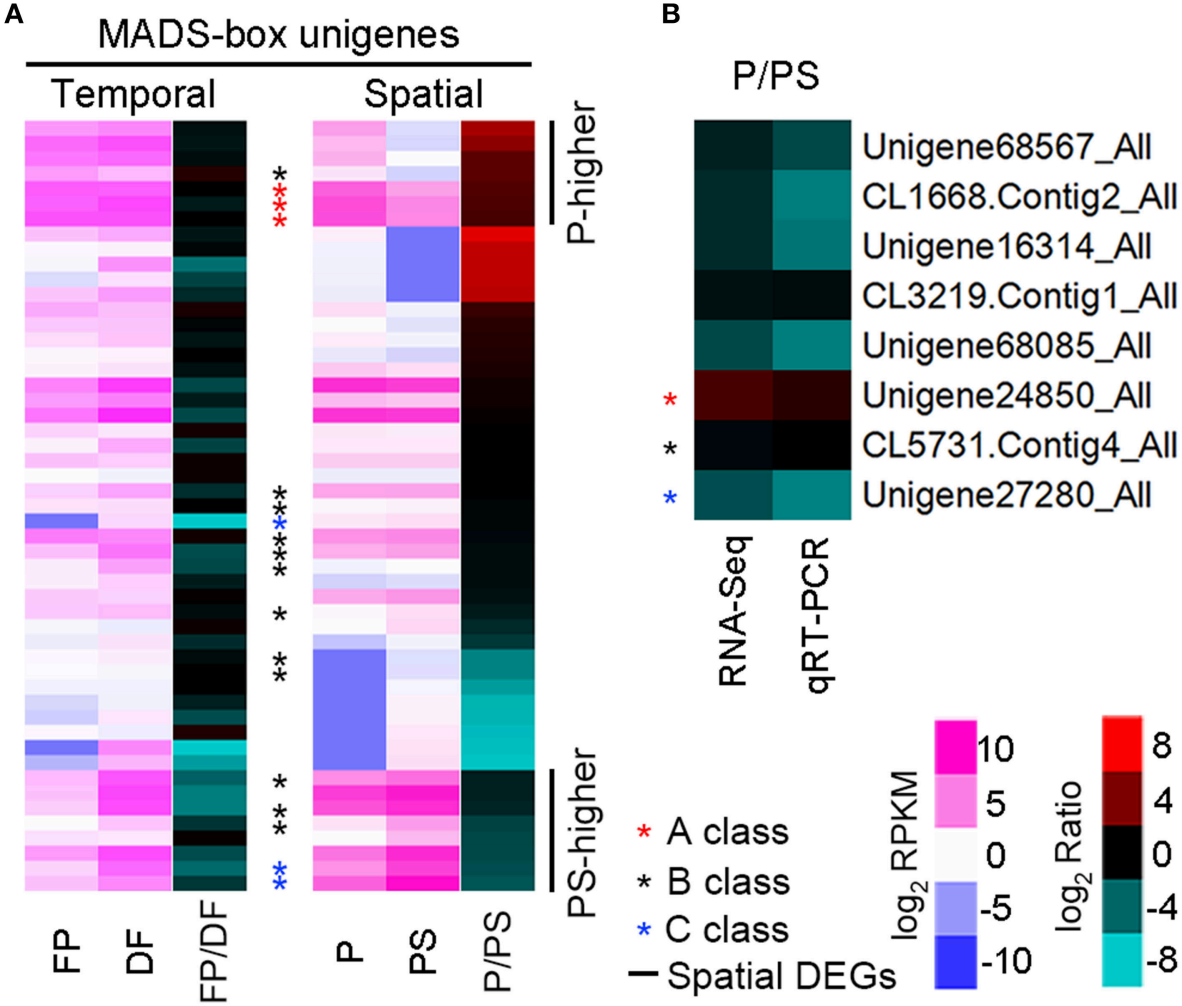
**Expression patterns of MADS-box and randomly selected unigenes. (A)** Heatmap illustrating the expression of 51 MADS-box unigenes. The left and middle columns show the RPKM value in P and PS respectively, and the right column represents the ratio of RPKM (P/PS). Asterisks indicate the putative A, B, and C class unigenes, and 7 P-higher and 8 PS-higher DEGs are lined out. **(B)** P/PS expression ratio from RNA-Seq (left) and qRT-PCR (right) for 8 unigenes.

According to the Nr annotation, 106 unigenes were identified as MADS-box family members, and 51 were detected in one or both organs of P and PS. We focused on these 51 unigenes, and analyzed the temporal-spatial expressions of them. 37 (72.5%) of them were expressed higher in DF than in FP, while 27 (52.9%) were expressed higher in PS than in P (Supplementary Table 3; Figure 6A). It seemed that there was a temporal expression preference for these MADS-box unigenes in DF, while no evident spatial preference was found in P or PS. Besides, 18 MADS-box unigenes were annotated as putative homologs of the ABC genes. Spatial expression analysis revealed that, all of the 3 A-class unigenes were P-higher DEGs, while 2 of 3 C-class *AG* homologs belonged to the PS-higher DEGs. The B-class unigenes exhibited intermediate expression patterns, with 1 P-higher, 3 PS-higher DEGs and 8 non-spatial-DEGs (Figure 6A). We selected 3 MADS-box unigenes *CiAP1-1, CiGLO-1*, and *CiAG-1* as the representative homologs of A-class gene *AP1*, B-class gene *GLO* and C-class gene *AG*, respectively, and validated their spatial expression patterns with quantitative real-time PCR (qRT-PCR), together with other 5 randomly selected unigenes (Figure 6B).

**Figure 7:**
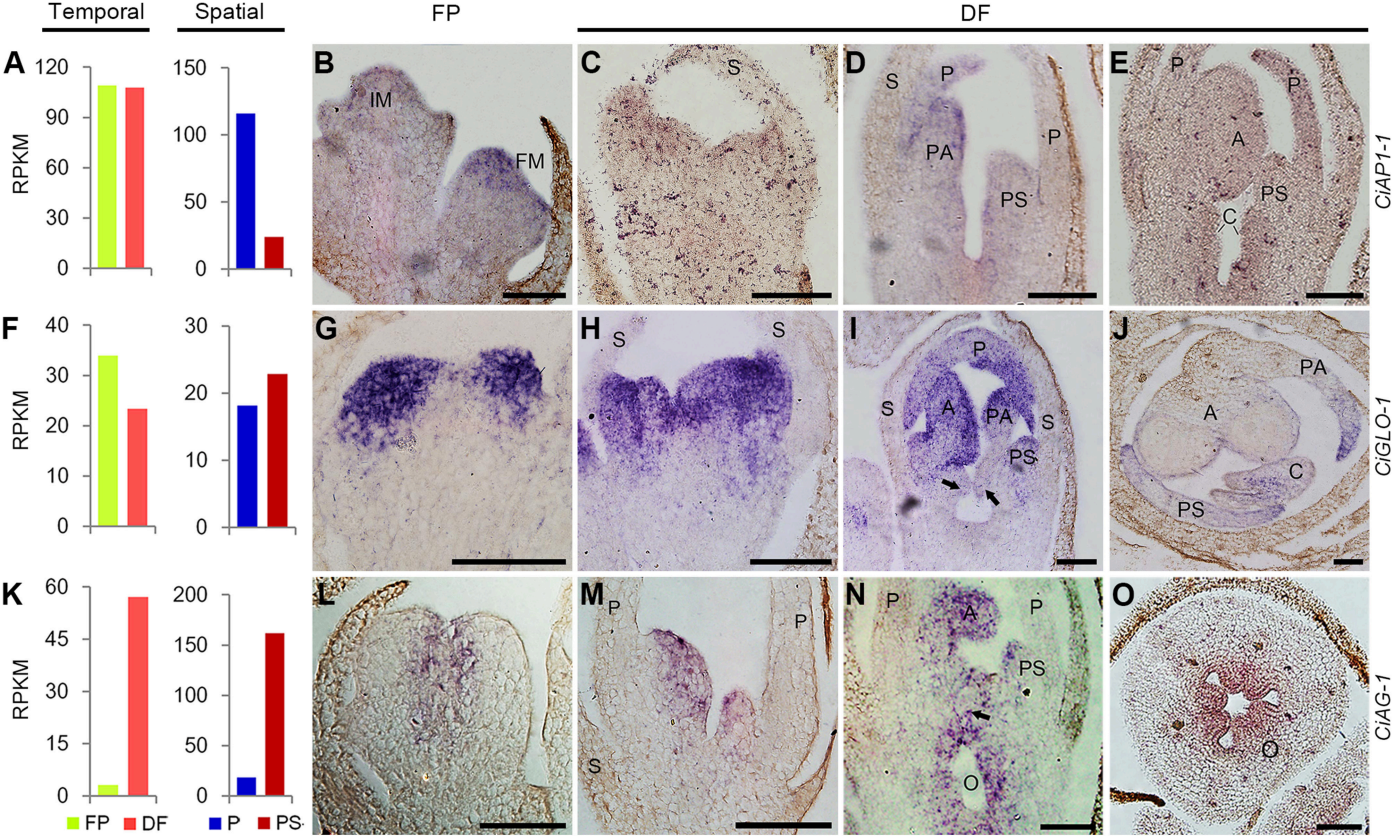
**Temporal-spatial expression of ABC unigenes during flower development. (A,F,K)**, RNA-Seq results showing the temporal and spatial expression of *CiAP1-1* **(A)**, *CiGLO-1* **(F)**, and *CiAG-1* **(K)**. All the others, *in situ* hybridization results of the temporal and spatial expression of *CiAP1-1* **(B–E)**, *CiGLO-1* **(G–J)**, and *CiAG-1* **(L–O)**. Arrows in **(I,N)** indicated the emerging carpel primordia. Sections in **(J,O)** were transverse and the others were all longitudinal. IM, inflorescence meristem; FM, flower meristem; S, sepal; P, petal; PS, petaloid staminode; PA, petaloid appendage; A, anther; C, carpel; O, ovary. Bars = 100 μm.

Based on the RNA-Seq results, the expression of *CiAP1-1* in FP and DF were almost the same, and *CiGLO-1* expression was moderately down-regulated in DF, while *CiAG-1* was remarkably up-regulated in DF. Spatially, *CiAP1-1* was expressed lower in PS than in P, *CiGLO-1* was expressed almost equally in the two types of organs, and *CiAG-1* was expressed much higher in PS than in P (Figures 7A,F,K). To define the expression patterns of these ABC genes more precisely, *in situ* hybridization was performed. *CiAP1-1* mRNA was detected in flower meristem, but not in inflorescence meristem (Figure 7B), showing a similar pattern to that in *Arabidopsis* (Mandel et al., 1992). At either early or late stage, *CiAP1-1* transcripts were detected in all whorls of the flower, except in sepals when they were comparatively old (Figures 7C–E). At early stages, *CiGLO-1* mRNA was detected in the edge of the floral cup (ring meristem) where petals and androecial members would develop, but not in the regions corresponding to sepals and carpels (Figure 7G). Later when petals and androecial members were established, *CiGLO-1* was expressed in both whorls, and no detectable signal was observed in the sepal and carpel primordia (Figures 7H,I). However, *CiGLO-1* mRNA was then detected in the young style, which mainly comprises the lateral carpel (Figure 7J). *CiAG-1* mRNA was firstly detected in the center of the floral meristem (Figure 7L), and then in all the androecial members, as well as in the carpels, while no expression signal was detected in sepals and petals (Figures 7M,N). *CiAG-1* also expressed in the ovary (Figure 7O). These results showed that the expression of A-function gene in *C. indica* is not confined to the first and second whorls, B-function gene could extend its expression domain to the gynoecium during development process, and C-function gene is expressed in the sterile petaloid staminodes as well as the fertile organs.

## Discussion

*Canna indica* is a representative species for studying phylogenetic evolution of Zingiberales. In this species, most of androecial members are transformed to petaloid organs and only a half fertile stamen remains in the flower. Little is known about the mechanism for the evolutionary conversion of stamens to petaloid organs, largely because the genomic information of *C. indica* is currently unavailable, and genetic transformation of this species is not applicable yet. With a large amount of transcript sequence data, our results provided fundamental information for studying the flower development of *C. indica*. Totally, 118,869 unigenes were sequenced in *C. indica*, among which 67,299 got annotated with gene names and functions. Meanwhile, 51,570 unigenes show no homology with the already known genes. Most of the annotated unigenes show the highest similarity with *M. acuminata* (Musaceae), which is also Zingiberales species, supporting the close genetic relationship between them.

Based on the (A)BC model, (A)-function genes specify the identity of sepal, which is the ground state of the flower (Causier et al., 2010). Expression analysis showed that A-class MADS-box unigenes in *C. indica* were all expressed higher in P than PS. Accordingly, it is inferred that petals possess more sepal identity than petaloid staminodes, and the similarities between petals and sepals could possibly support this supposition. The fact that *CiAP1-1* was expressed in all whorls reminded us of *CAULIFLOWER* (*CAL*), the closest paralogue of *AP1*, which is also expressed in all whorls at early stages (Kempin et al., 1995). However, the expression pattern of *CiAP1-1* in later stages was dissimilar to either *AP1* or *CAL* in *Arabidopsis*.

RNA-Seq and *in situ* hybridization results showed that C-function unigene *CiAG-1* was highly expressed in PS, while showed much lower or no expression in P. The combined expression of *CiGLO-1* and *CiAG-1* in the whorl 3 suggests that the petaloid staminodes are of androecial identity, which is in accordance with the traditional view, and with the case in *A. hainanensis* (Zingiberaceae), another Zingiberales species (Song et al., 2010). However, this evidence was not sufficient to explain the different fates of the androecial members since co-expression of B- and C-class genes specifies stamen identity (Fu et al., 2014).

Expansion of B-class domain to other whorls was widely observed in angiosperm including *Arabidopsis* and *Antirrhitum* (Zahn et al., 2005). In *C. indica*, transcripts of *CiGLO-1* were not detected in the carpel primordia at early stages, while were detectable in the style primordium later. It seems that B-class is not involved in initiation and identity determination of carpels, while plays a role in the partially petaloid morphogenesis of the style (Glinos and Cocucci, 2011; Fu et al., 2014).

In the flowers of *C. indica*, petals turn partially sepaloid, while members of the inner whorls including staminodes, fertile stamen and style are petaloid more or less. Taken all these homeotic changes into consideration, we speculated that the sepal and petal identities have shifted inwards uniformly in Canna flowers. In accordance with this supposition, the expressions of both A- and B-function genes are expanded to inner whorls to some extent. However, the remaining expression of C-function genes in petaloid androecium members makes it complicated to explain the stamen-to-petal conversion of these organs. Besides expression domain, the sequence diversity of C-function genes may also relate to the androecial petaloidy in Zingiberales (Almeida et al., 2015). To eventually reveal the exact mechanism for this kind of homeotic conversion, much more work is needed.

To summarize, through RNA-Seq and *in situ* hybridization analyses, we found a correlation between expression patterns of ABC genes and the organ identities of floral organs. A-function gene is expressed in sepals, A- and B-function genes are expressed in petals, A-, B-, and C-function genes are expressed in androecial members, A- and C-function genes are expressed in carpels at earlier stages, and later, B-function gene also appears in the style. Although, the mechanism for the differentiation of androecial members remains unclear, the current expression results as well as the sequences data have provided a preliminary basis for further researches on flower development in *C. indica*, and on the phylogenetic development of Zingiberales.

## Accession numbers

All the RNA-seq raw reads were deposited in Gene Expression Omnibus (GEO) (Accession GSE72440 and GSE72441).

## Author contributions

HL and JL conceived and designed the research. XT performed the RNA-Seq, qRT-PCR, and *in situ* hybridization experiments, QY performed the SEM observation. XT wrote the manuscript and all authors contributed to the discussion and manuscript revision.

### Conflict of interest statement

The authors declare that the research was conducted in the absence of any commercial or financial relationships that could be construed as a potential conflict of interest. The reviewer XS and handling Editor declared their shared affiliation, and the handling Editor states that the process nevertheless met the standards of a fair and objective review.
