# Corrigendum: Temporal-Spatial Transcriptome Analyses Provide Insights into the Development of Petaloid Androecium in *Canna indica*

**DOI:** 10.3389/fpls.2016.01745

**Published:** 2016-11-15

**Authors:** Xueyi Tian, Qianxia Yu, Huanfang Liu, Jingping Liao

**Affiliations:** ^1^Key Laboratory of Plant Resources Conservation and Sustainable Utilization, South China Botanical Garden, Chinese Academy of SciencesGuangzhou, China; ^2^College of Life Sciences, University of Chinese Academy of SciencesBeijing, China

**Keywords:** transcriptome, RNA-Seq, petaloid staminode, Zingiberales, *Canna indica*, ABC model

Due to an oversight, the accession numbers of the RNA-seq raw data in the public repository were inadvertently missed in the original article. The authors would like to add the following statement to this article:

All the RNA-seq raw reads were deposited in Gene Expression Omnibus (GEO) (Accession GSE72440 and GSE72441).

This change does not affect the scientific conclusions of the article in any way. The authors apologize for this oversight.

The original article has been updated.

## Conflict of interest statement

The authors declare that the research was conducted in the absence of any commercial or financial relationships that could be construed as a potential conflict of interest.

